# Interpregnancy Interval After Healthy Live Birth and Subsequent Spontaneous Abortion

**DOI:** 10.1001/jamanetworkopen.2024.17397

**Published:** 2024-06-17

**Authors:** Xuan Hu, Ying Yang, Long Wang, Chuanyu Zhao, Xinyi Lyu, Meiya Liu, Hanbin Wu, Jueming Lei, Jiaxin Li, Mengxin Yao, Yaling Ding, Hongguang Zhang, Yuan He, Yuanyuan Wang, Zuoqi Peng, Haiping Shen, Qiaomei Wang, Yiping Zhang, Donghai Yan, Jieyun Yin, Xu Ma

**Affiliations:** 1 National Research Institute for Family Planning, Beijing, China; 2 Department of Epidemiology and Health Statistics, School of Public Health, Medical College of Soochow University, Suzhou, Jiangsu, China; 3 National Human Genetic Resources Center, Beijing, China; 4 Graduate School of Peking Union Medical College, Beijing, China; 5 Institute of Epidemiology and Statistics, School of Public Health, Lanzhou University, Lanzhou, China; 6 Department of Maternal and Child Health, National Health Commission of the People’s Republic of China, Beijing, China; 7 Jiangsu Key Laboratory of Preventive and Translational Medicine for Major Chronic Non-communicable Diseases, Soochow University, Jiangsu, China; 8 MOE Key Laboratory of Geriatric Diseases and Immunology, Suzhou Medical College of Soochow University, Suzhou, Jiangsu, China

## Abstract

**Importance:**

Many studies have reported that the interpregnancy interval (IPI) is a potential modifiable risk factor for adverse perinatal outcomes. However, the association between IPI after live birth and subsequent spontaneous abortion (SA) is unclear.

**Objective:**

To investigate the association of IPI after a healthy live birth and subsequent SA.

**Design, Setting, and Participants:**

This prospective cohort study used data from 180 921 women aged 20 to 49 years who had a single healthy live birth and planned for another pregnancy and who participated in the Chinese National Free Prepregnancy Checkups Project from January 1, 2010, to December 31, 2020. Statistical analysis was conducted from June 20 to October 5, 2023.

**Exposure:**

Interpregnancy interval, defined as the interval between the delivery date and conception of the subsequent pregnancy, was categorized as follows: less than 18 months, 18 to 23 months, 24 to 35 months, 36 to 59 months, and 60 months or longer.

**Main Outcomes and Measures:**

The main outcome was SA. Multivariable-adjusted odds ratios (ORs) were calculated by logistic regression models to examine the association between IPI and the risk of SA. Dose-response associations were evaluated by restricted cubic splines.

**Results:**

The analyses included 180 921 multiparous women (mean [SD] age at current pregnancy, 26.3 [2.8] years); 4380 SA events (2.4% of all participants) were recorded. A J-shaped association between IPI levels and SA was identified. In the fully adjusted model, compared with IPIs of 18 to 23 months, both short (<18 months) and long (≥36 months) IPIs showed an increased risk of SA (IPIs of <18 months: OR, 1.15 [95% CI, 1.04-1.27]; IPIs of 36-59 months: OR, 1.28 [95% CI, 1.15-1.43]; IPIs of ≥60 months: OR, 2.13 [95% CI, 1.78-2.56]). Results of the subgroup analysis by mode of previous delivery were consistent with the main analysis.

**Conclusions and Relevance:**

This cohort study of multiparous women suggests that an IPI of shorter than 18 months or an IPI of 36 months or longer after a healthy live birth was associated with an increased risk of subsequent SA. The findings are valuable to make a rational prepregnancy plan and may facilitate the prevention of SA and improvement in neonatal outcomes.

## Key Points

**Question:**

What is the association between interpregnancy interval (IPI) after a healthy live birth and subsequent spontaneous abortion (SA)?

**Findings:**

In this population-based cohort study of 180 921 Chinese women, both short (<18 months) or long (≥36 months) IPIs after a healthy live birth were associated with a higher risk of subsequent SA.

**Meaning:**

The findings of this study provide a scientific reference for prepregnancy planning, SA prevention, and maternal and fetal health improvement.

## Teaser

This cohort study investigates the association between the interpregnancy interval after a healthy live birth and subsequent spontaneous abortion among more than 180 000 women in China.

## Introduction

Spontaneous abortion (SA), also called miscarriage, can result in a variety of short-term obstetrical complications, including recurrent SA, premature birth, and placental abruption,^1^ and is associated with long-term health problems, such as cardiovascular disease and venous thromboembolism.^2^ Because SA not only negatively affects individuals’ health but also places a burden on family members and the health care system, identifying risk factors associated with SA is crucial.

Over half of all SAs, especially recurrent SAs, are associated with chromosomal abnormalities,^3^ and the rest can be attributed to risk factors such as older age, passive smoking, chronic maternal diseases, previous SA, air pollution,^2,4^ and some unknown reasons. Most of these factors are usually difficult for individuals to change. Some scholars have proposed that the interpregnancy interval (IPI) is a potential modifiable risk factor for adverse pregnancy outcomes,^5,6,7^ and previous evidence has shown that a short IPI after an abortion may reduce the risk of a subsequent SA.^8,9^ Nevertheless, the association between IPI after live birth and subsequent SA is less studied and remains unclear, particularly among parous women with healthy live births. The association of IPI after live birth with subsequent adverse perinatal outcomes has been widely reported.^6,10,11^ The IPI was considered optimal if the pregnancy occurred at 18 to 23 months^12,13^ or 12 to 23 months^10,14^ after the previous live birth. Both short IPIs and extraordinarily long IPIs are strongly associated with a higher risk of preterm birth, low birth weight, and small size for gestational age.^11,12,13^ Biologically, short IPIs along with maternal nutritional depletion, unresolved inflammation,^15^ and incomplete uterine recovery^16^ and long IPIs alongside possible physiological regression^17^ are both demonstrated to be involved in the pathogenesis of SA. Therefore, we hypothesized that SA during pregnancy may be associated with short IPIs and long IPIs.

In the present study, we conducted a national population-based cohort study in Chinese reproductive-aged women to elucidate the association between IPI after a healthy live birth and subsequent SA. Exploring appropriate IPIs is essential for the health of both the mother and the fetus to prevent SA and further improve maternal and fetal health.

## Methods

### Data Sources and Study Design

This cohort study used data from the National Free Prepregnancy Checkups Project (NFPCP). The NFPCP, initiated by the National Health Commission and Ministry of Finance of the People’s Republic of China, aims to offer free preconception health examinations and follow-up of pregnancy outcomes for reproductive-aged couples. Preconception baseline information, including demographic characteristics, reproductive history, disease history, and lifestyle, was collected by health staff in the local maternal and child health care service centers by using a face-to-face standard and structured questionnaire followed by the preconception health examination. All participants were followed up by trained local health workers by telephone for early pregnancy and pregnancy outcomes. The project-related design, organization, and implementation of the NFPCP have been described previously.^18,19^ The study was conducted according to the guidelines of the Declaration of Helsinki^20^ and approved by the institutional research review board at the National Health and Family Planning Commission, now known as the National Health Commission. All participants provided written informed consent before enrollment. This study followed the Strengthening the Reporting of Observational Studies in Epidemiology (STROBE) reporting guideline.

**Figure 1.**
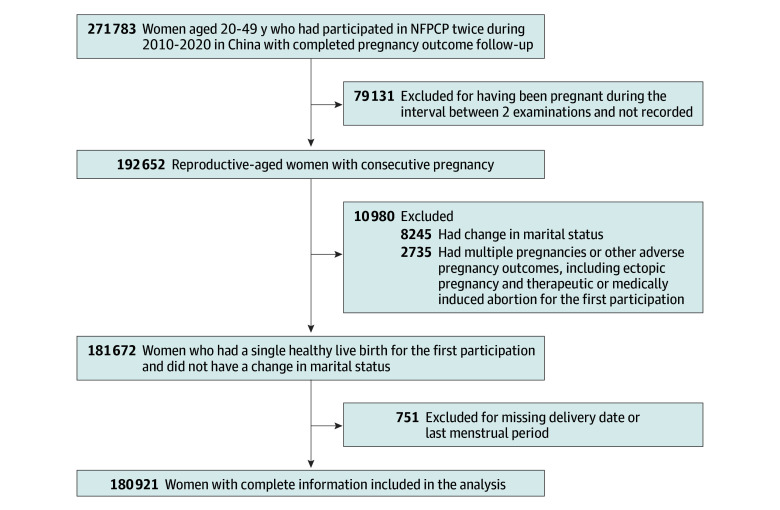
Flowchart of the Study Population NFPCP indicates National Free Prepregnancy Checkups Project.

Because China has implemented a 2-child and 3-child policy^21,22^ in succession from November 2015, the NFPCP also supports repeat participation for women who wish to have a second or third child. Therefore, based on NFPCP registration data, 271 783 women aged 20 to 49 years who had participated in the NFPCP twice between January 1, 2010, and December 31, 2020, with completed pregnancy outcome follow-up, were enrolled in the study. If the difference between the reported number of previous pregnancies in 2 prepregnancy examinations was greater than 1, it was considered a nonconsecutive pregnancy. To calculate the IPI more accurately, we excluded such participants (n = 79 131). In addition, other exclusion criteria comprised women with changes in spouse (n = 8245), those with multiple pregnancies or other adverse pregnancy outcomes (including premature birth, macrosomia, low birth weight, large size for gestational age, small size for gestational age, SA, or stillbirth) for the first participation (n = 2735), and those lacking information on delivery dates or last menstrual period (LMP) data (n = 751). A total of 180 921 women were included in the present analysis. Detailed information on the study population recruitment and derivation is shown in Figure 1.

### Exposure

Interpregnancy interval, defined as the interval between the delivery date and conception of the subsequent pregnancy, is calculated by subtracting the previous delivery date from the current LMP. Within 3 months of the baseline examination, researchers conducted an initial interview to follow up on the participants’ pregnancy status and record their LMP. For women with successful pregnancies, local health workers conducted several follow-up telephone interviews within 1 year from the initial visit to gather delivery-related information. The results and date of each follow-up visit were recorded until discontinuation after a definitive pregnancy outcome (live birth, SA, or stillbirth). Interpregnancy intervals were stratified into 5 groups in the main models: group 1, shorter than 18 months; group 2, 18 to 23 months (reference group); group 3, 24 to 35 months; group 4, 36 to 59 months; and group 5, 60 months or longer (eAppendix and eTable 1 in Supplement 1).

### Outcomes

The main outcome was SA, defined as fetal death or pregnancy loss, occurring before 28 weeks of gestation. Information on SA was obtained from participants’ self-reports. Trained local health personnel followed up via telephone with women who had successfully conceived to collect information on pregnancy outcomes, encompassing details of mode of delivery (cesarean or vaginal delivery), fetal outcome, and number of fetuses.

### Covariables

The covariables used in model adjustment include maternal age at LMP (<30 years or ≥30 years), prepregnancy body mass index (BMI; calculated as weight in kilograms divided by height in meters squared) (<18.5, 18.5-23.9, 24.0-27.9, or ≥28.0), mode of delivery at previous birth (cesarean or vaginal delivery), maternal educational level (junior high school or below or high school or above), ethnicity (Han nationality or any of 55 other ethnic minority groups in China), alcohol consumption (yes or no), smoking (yes or no), and history of any abortion (yes or no). Covariance diagnostics were performed on all variables, and no collinearity issues were found based on the variance inflation factor and tolerance.

### Statistical Analysis

Statistical analysis was conducted from June 20 to October 5, 2023. All variables were described as categorical variables, expressed as numbers and percentages. The χ^2^ test was used to compare the distributions of baseline characteristics among the different IPI groups. To allow for curvilinear shapes, we also modeled IPI as a continuous variable and graphed the adjusted odds ratios (ORs) using restricted cubic splines with 3 knots.

The multivariable-adjusted ORs were calculated by logistic regression models to examine the association between IPI with the risk of SA. We fitted 2 logistic regression models with a priori–selected covariables. The crude OR was calculated by an unadjusted logistic regression. The adjusted OR was adjusted for maternal age at LMP, prepregnancy BMI, mode of previous delivery, alcohol consumption, smoking, history of abortion, maternal educational level, and ethnicity.

Stratified and subgroup analyses were conducted according to the mode of previous delivery and maternal age. In addition, a sensitivity analysis was also performed by excluding women with a history of abortion. In some countries, SA is defined as a loss before 20 weeks of pregnancy.^23,24^ Considering that the pathogenesis of SA may differ at different gestational ages, we also categorized SA into 2 categories based on follow-up record dates: less than 20 weeks and 20 to 28 weeks.

All analyses were performed using R, version 4.2.2 (R Project for Statistical Computing), with the rms, ggplot2, forest, speedglm, and nnet packages. A 2-sided *P* < .05 was considered statistically significant.

## Results

A total of 180 921 multiparous women (mean [SD] age at current pregnancy, 26.3 [2.8] years) were included (Table 1). Among all participants, 67 647 (37.4%) had an IPI shorter than 18 months, 29 278 (16.2%) had an IPI of 18 to 23 months, 45 670 (25.2%) had an IPI of 24 to 35 months, 34 553 (19.1%) had an IPI of 36 to 59 months, and 3773 (2.1%) had an IPI of 60 months or longer. The mean (SD) maternal age at previous delivery was 23.5 (2.5) years. Table 1 shows that, compared with women with an IPI of 18 to 23 months, women with a longer IPI were more likely to be older, more educated, and have a higher BMI.

**Table 1.** Characteristics of the Participants According to Interpregnancy Interval

Maternal Characteristic	Total (N = 180 921)	Interpregnancy interval, mo					
		<18 (n = 67 647)	18-23 (n = 29 278)	24-35 (n = 45 670)	36-59 (n = 34 553)	≥60 (n = 3773)	*P* value
Age at previous delivery, mean (SD), y	23.5 (2.5)	23.4 (2.5)	23.5 (2.5)	23.5 (2.5)	23.4 (2.5)	23.2 (2.4)	<.001
Age at previous delivery, No. (%)							
<30 y	176 673 (97.7)	66 092 (97.7)	28 592 (97.7)	44 524 (97.5)	33 744 (97.7)	3721 (98.6)	.001
≥30 y	4248 (2.3)	1555 (2.3)	686 (2.3)	1146 (2.5)	809 (2.3)	52 (1.4)	
Age at current LMP, mean (SD), y	26.3 (2.8)	25.1 (2.6)	26.0 (2.5)	26.7 (2.5)	27.8 (2.5)	29.4 (2.4)	<.001
Age at current LMP, No. (%)							
<30 y	160 056 (88.5)	64 003 (94.6)	26 851 (91.7)	40 016 (87.6)	27 022 (78.2)	2164 (57.4)	<.001
≥30 y	20 865 (11.5)	3644 (5.4)	2427 (8.3)	5654 (12.4)	7531 (21.8)	1609 (42.6)	
Educational level, No. (%)							
Junior high school or below	153 155 (84.7)	58 132 (85.9)	24 704 (84.4)	38 312 (83.9)	28 894 (83.6)	3113 (82.5)	<.001
High school or above	22 147 (12.2)	7013 (10.4)	3600 (12.3)	6053 (13.3)	4887 (14.1)	594 (15.7)	
Missing data	5619 (3.1)	2502 (3.7)	974 (3.3)	1305 (2.9)	772 (2.2)	66 (1.8)	
Prepregnancy BMI, No. (%)							
Underweight (<18.5)	19 109 (10.6)	8054 (11.9)	3340 (11.4)	4344 (9.5)	3034 (8.8)	337 (8.9)	<.001
Normal weight (18.5-23.9)	125 632 (69.4)	47 574 (70.3)	20 113 (68.7)	31 498 (69.0)	23 835 (69.0)	2612 (69.2)	
Overweight (24.0-27.9)	7285 (4.0)	2113 (3.1)	1187 (4.1)	2100 (4.6)	1694 (4.9)	191 (5.1)	
Obese (≥28.0)	28 679 (15.9)	9814 (14.5)	4602 (15.7)	7694 (16.8)	5942 (17.2)	627 (16.6)	
Missing data	216 (0.1)	92 (0.1)	36 (0.1)	34 (0.1)	48 (0.1)	6 (0.2)	
Mode of previous delivery, No. (%)							
Cesarean delivery	39 151 (21.6)	8765 (13.0)	5774 (19.7)	11 901 (26.1)	11 126 (32.2)	1585 (42.0)	.52
Vaginal delivery	141 566 (78.3)	58 849 (87.0)	23 496 (80.3)	33 735 (73.9)	23 342 (67.6)	2144 (56.8)	
Missing data	204 (0.1)	33 (0.05)	8 (0.03)	34 (0.1)	85 (0.2)	44 (1.2)	
Smoking, No. (%)							
No	180 414 (99.7)	67 470 (99.7)	29 195 (99.7)	45 537 (99.7)	34 454 (99.7)	3758 (99.6)	.53
Yes	125 (0.1)	43 (0.1)	22 (0.1)	26 (0.1)	29 (0.1)	5 (0.1)	
Missing data	382 (0.2)	134 (0.2)	61 (0.2)	107 (0.2)	70 (0.2)	10 (0.3)	
Alcohol consumption, No. (%)							
No	178 530 (98.7)	66 973 (99.0)	28 946 (98.9)	45 043 (98.6)	33 924 (98.2)	3644 (96.6)	<.001
Yes	1598 (0.9)	443 (0.7)	231 (0.8)	444 (1.0)	426 (1.2)	54 (1.4)	
Missing data	793 (0.4)	231 (0.3)	101 (0.3)	183 (0.4)	203 (0.6)	75 (2.0)	
Ethnicity, No. (%)							
Han	168 597 (93.2)	62 326 (92.1)	27 287 (93.2)	42 920 (94.0)	32 484 (94.0)	3580 (94.9)	<.001
Other^a^	3380 (1.9)	1213 (1.8)	451 (1.5)	741 (1.6)	855 (2.5)	120 (3.2)	
Missing data	8944 (4.9)	4108 (6.1)	1540 (5.3)	2009 (4.4)	1214 (3.5)	73 (1.9)	
History of any abortion, No. (%)							
No	170 020 (94.0)	64 038 (94.7)	27 653 (94.4)	42 822 (93.8)	32 041 (92.7)	3466 (91.9)	<.001
Yes	8642 (4.8)	2791 (4.1)	1314 (4.5)	2302 (5.0)	1984 (5.7)	251 (6.7)	
Missing data	2259 (1.2)	818 (1.2)	311 (1.1)	546 (1.2)	528 (1.5)	56 (1.5)	

Abbreviations: BMI, body mass index (calculated as weight in kilograms divided by height in meters squared); LMP, last menstrual period.

^a^ Including 55 ethnic minority groups in China.

A total of 4380 SA events were recorded in this study, which accounted for 2.4% of all participants. The proportion of SAs was 2.3% (1587 of 67 647) among women with an IPI shorter than 18 months, 2.1% (615 of 29 278) among women with an IPI of 18 to 23 months, 2.3% (1034 of 45 670) among women with an IPI of 24 to 35 months, 2.8% (961 of 34 553) among women with an IPI of 36 to 59 months, and 4.9% (183 of 3773) among women with an IPI of 60 months or longer (Table 2). A significant J-shaped association was found between continuous IPI variables and SA risk. The intersection of the curve with an OR of 1 corresponds to IPI values of approximately 21.7 and 24.6 months (eFigure 1 in Supplement 1). Therefore, in the next analyses, we considered an IPI of 18 to 23 months, which was also proposed by other studies,^7,11^ as the reference group.

**Table 2.** Risks of Spontaneous Abortion According to IPI in China From 2010 to 2020

IPI, mo	Spontaneous abortion, No./total No. (%)	OR (95% CI)	
		Crude	Adjusted^a^
<18	1587/67 647 (2.3)	1.12 (1.02-1.23)	1.15 (1.04-1.27)
18-23	615/29 278 (2.1)	1.00 [Reference]	1.00 [Reference]
24-35	1034/45 670 (2.3)	1.08 (0.98-1.19)	1.05 (0.95-1.17)
36-59	961/34 553 (2.8)	1.33 (1.20-1.48)	1.28 (1.15-1.43)
≥60	183/3773 (4.9)	2.38 (2.01-2.81)	2.13 (1.78-2.56)

Abbreviations: IPI, interpregnancy interval; OR, odds ratio.

^a^ Adjusted for maternal age at last menstrual period, prepregnancy body mass index, mode of previous delivery, alcohol consumption, smoking, history of abortion, maternal educational level, and ethnicity.

In the fully adjusted model, compared with the reference group (IPI of 18-23 months), those with an IPI of shorter than 18 months exhibited a 15% higher risk of SA (OR, 1.15 [95% CI, 1.04-1.27]) (Table 2). Furthermore, an IPI of 36 to 59 months had a 28% higher SA risk (OR, 1.28 [95% CI, 1.15-1.43]), whereas an IPI of 60 months or longer significantly increased the risk of SA by 113% (OR, 2.13 [95% CI, 1.78-2.56]). After adjustment for various covariables, these estimates were similar across all models. However, there was no significant association found between an IPI of 24 to 35 months and SA (adjusted OR, 1.05 [95% CI, 0.95-1.17]). Therefore, we also adopted an IPI of 18 to 35 months as a reference group and found that the association of an IPI of shorter than 18 months with SA was no longer statistically significant (adjusted OR, 1.05 [95% CI, 0.97-1.13]) (eTable 3 in Supplement 1). Similar results were observed in the sensitivity analysis after excluding participants with a history of abortion (eTable 2 in Supplement 1).

**Figure 2.**
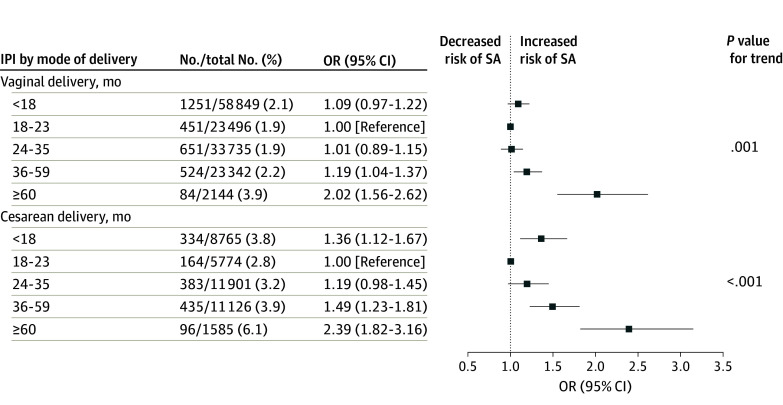
Risks of Spontaneous Abortion (SA) According to Mode of Previous Delivery by Interpregnancy Interval (IPI) in China From 2010 to 2020 Odd ratios (ORs) were fully adjusted by maternal age at last menstrual period, prepregnancy body mass index, mode of previous delivery, alcohol consumption, smoking, history of abortion, maternal educational level, and ethnicity. The reference group was women who had an IPI of 18 to 23 months.

In a subgroup analysis stratified by mode of previous delivery, the trend of estimates was generally consistent with the main analysis (Figure 2). To discover the combined associations of IPI and prior modes of delivery with SA, the OR was recalculated using an IPI of 18 to 23 months after vaginal delivery as the reference group. Women with an IPI 36 months or longer or whose last birth was a cesarean delivery had a significantly increased risk of SA. Women whose IPI was 60 months or longer after a cesarean delivery exhibited the highest risk of SA (adjusted OR, 3.52 [95% CI, 2.78-4.45]) (Table 3; eFigure 3 in Supplement 1). Meanwhile, within the same IPI group, cesarean delivery always yielded a higher risk of subsequent SA than vaginal delivery. After setting the reference group as an IPI of 18 to 35 months, the association of IPIs shorter than 18 months with SA in the vaginal delivery group became significant (eTable 4 in Supplement 1).

**Table 3.** Association Between the IPI After Different Mode of Delivery and the Risk of Spontaneous Abortion in China From 2010 to 2020

IPI, mo^b^	Crude OR (95% CI)		Adjusted OR (95% CI)^a^	
	Vaginal delivery	Cesarean delivery	Vaginal delivery	Cesarean delivery
<18	1.11 (1.00-1.24)	2.02 (1.75-2.34)	1.09 (0.97-1.22)	1.99 (1.71-2.31)
18-23	1.00 [Reference]	1.49 (1.25-1.79)	1.00 [Reference]	1.47 (1.21-1.78)
24-35	1.01 (0.89-1.14)	1.70 (1.48-1.95)	1.00 (0.88-1.14)	1.75 (1.51-2.02)
36-59	1.17 (1.03-1.33)	2.08 (1.82-2.38)	1.19 (1.04-1.36)	2.19 (1.90-2.52)
≥60	2.08 (1.64-2.64)	3.29 (2.63-4.13)	2.05 (1.59-2.65)	3.52 (2.78-4.45)

Abbreviations: IPI, interpregnancy interval; OR, odds ratio.

^a^ Adjusted for maternal age at last menstrual period, prepregnancy body mass index, mode of previous delivery, alcohol consumption, smoking, history of abortion, maternal educational level, and ethnicity.

^b^ A total of 204 participants were not included due to a lack of data on mode of delivery.

Considering that the definition of SA in some countries was before 20 weeks’ gestational age,^23,24^ SA was then separated into less than 20 weeks and 20 to 28 weeks. An IPI of 36 months or longer was associated with an increased risk of pregnancy loss, either occurring in the early stage (<20 weeks) or later stage (20-28 weeks) of pregnancy. In contrast, an IPI of shorter than 18 months was significantly associated with the risk of pregnancy loss only between 20 and 28 weeks’ gestational age (eTable 5 in Supplement 1). Moreover, when IPIs of 18 to 35 months were taken as the reference group, the results did not change significantly (eTable 6 in Supplement 1).

According to the age at previous delivery, we also stratified the participants to younger than 30 years and 30 years or older. In each IPI stratum, women who previously experienced childbirth at 30 years of age or older always had a higher risk of subsequent SA than those whose last childbirth occurred before 30 years of age. Meanwhile, the IPI range of 24 to 35 months was significantly associated with increased risk of subsequent SA among women who previously experienced childbirth at 30 years of age or older (adjusted OR, 3.05 [95% CI, 1.16-8.02]) but not among those whose last childbirth occurred before 30 years of age (adjusted OR, 1.04 [95% CI, 0.93-1.15]) (eFigure 2 in Supplement 1). These results suggest that a longer IPI is associated with more hazardous outcomes among older women.

## Discussion

To our knowledge, this population-based cohort study is the first analysis to explore the association between IPI after a healthy live birth and the risk of subsequent SA. It has been reported that previous adverse birth outcomes may influence subsequent pregnancy.^25^ In comparison with previous relevant studies,^5,11^ women who experienced live adverse outcomes in prior deliveries (such as premature birth or macrosomia) were additionally excluded to better assess the association of IPI with outcomes. In the present study, a J-shaped association between IPI after a healthy live birth and subsequent SA risk was observed. After adjusting for multiple covariables, both short (<18 months) and long (≥36 months) IPIs were significantly associated with increased risk of SA. Furthermore, we also discovered that women who had subsequent pregnancies after cesarean delivery or were 30 years of age or older during their last childbirth were more likely to experience SA in their next pregnancy.

The main finding in this study is that increased risks of SA during pregnancies were observed among women with an IPI shorter than 18 months or 36 months or longer after a healthy live birth. Several studies have shown that a short IPI after an SA does not increase the risk of recurrent SAs.^8,9,26^ However, to our knowledge, no previous study had been conducted to illustrate the association between IPI after live birth and subsequent SA. Maternal health characteristics after a healthy live birth differed from those after SA. Unlike live births, an SA is less likely to lead to folate deficiency in the postpartum period and is more favorable for embryonic development in a subsequent pregnancy.^9,27^ One study performed stratified analyses by previous pregnancy outcomes and found that a short IPI after live birth, but not after stillbirth or SA, resulted in a significantly increased risk of perinatal mortality in subsequent pregnancies.^26^ Previous epidemiologic studies have demonstrated a significant association between a short or very long IPI after live birth and increased risk of preterm birth, low birth weight, and small size for gestational age.^6,12^ We consistently found that the IPI after a healthy live birth and risk of subsequent SA also align with this trend. Taking other studies focused on IPI and other adverse pregnancy outcomes into consideration may provide more comprehensive and scientific guidance for women’s fertility planning.

In the subgroup analysis, our analysis revealed that cesarean delivery, including a short IPI, may be more prone to subsequent SA. This finding is in line with a previous study.^11^ Cesarean delivery can lead to endometrial thinning at the uterine scar, potentially resulting in partial or complete loss of the basal decidua. In addition, the chorionic tissue may invade the myometrium,^28^ which requires sufficient time for recovery to prevent compromised fertility. Bujold and Gauthier^29^ believed that an IPI shorter than 18 months should be considered a risk factor for uterine rupture for women who have had a previous cesarean delivery, which supports our findings to some extent.

In the analysis stratified by age at previous delivery, we found that women aged 30 years or older during both previous and current pregnancies were more vulnerable to subsequent SA. This finding is consistent with the study by Nybo Andersen et al,^30^ which concluded that the risk of SA was significantly increased for women older than 30 years of age, regardless of parity and reproductive history. Similar findings have been reported for other adverse outcomes, such as maternal death and stillbirth.^31^ In many countries, women are having their first child at an older age.^32^ Therefore, it is important to carefully consider the recommendations for future pregnancies in light of the potential risks associated with advancing maternal age.

Currently, the potential factors associated with SA remain incompletely understood.^33^ The role that a short IPI plays in SA after healthy live births may be explained by a few different reasons. One is that short intervals between pregnancies can lead to maternal nutritional depletion.^34^ For example, folate depletion can impede embryonic development in midpregnancy by preventing collagen cross-linking and weakening tissue connection.^16^ It takes about 1 year for maternal folate levels to revert to standard concentrations after childbirth.^35^ Another reason is that a short IPI is detrimental to the recovery of uterine inflammation and contraction-related proteins^36^ and therefore is susceptible to uterine rupture and uteroplacental bleeding disorders, especially after cesarean delivery.^37^

In some countries, SA is defined as intrauterine death at less than 20 weeks of gestation.^23,24^ Therefore, sensitivity analyses were conducted by categorizing SA into 2 stages: before 20 weeks and from 20 to 28 weeks. The findings showed that women with an IPI 36 months or longer had an increased risk of both SA before 20 weeks and SA between 20 and 28 weeks. An IPI shorter than 18 months was associated with increased risks of SA between 20 and 28 weeks but was not statistically significant for SA before 20 weeks. Less than one-third of total SAs occurred before 20 weeks in our study. An early SA is difficult to detect because it can easily be mistaken for delayed menstruation. Therefore, these results related to SA occurring before 20 weeks in this study should be interpreted and applied with caution, as more future prospective cohort studies are needed to verify these findings.

Given that long IPIs are frequently observed among women who conceive at an older age, it is imperative to investigate whether the association of long IPIs with SA is due primarily to maternal age. After adjusting for current maternal pregnancy age, an IPI of 36 months or longer remained significantly associated with SA; similar results were obtained in the subgroup by age at previous delivery. Therefore, we conclude that long IPIs are an independent risk factor for SA. The physiologic regression hypothesis proposes that pregnancy can assist women in developing growth-supporting capacities, such as enhanced uterine blood flow and other physiological and anatomical adaptations of the reproductive system, to cater to the demand of placental and fetal growth, but these capacities are gradually diminished as IPI extends.^17^

Our data show that, after a healthy birth, an IPI of shorter than 18 months or an IPI of 36 months or longer was associated with the risk of subsequent SA. Cesarean delivery and advanced maternal age also appeared to be deleterious. Further high-quality studies are needed to explore the association between IPI and SA and to examine the underlying mechanisms.

### Limitations

Several limitations should be considered when the results of this study are interpreted. First, although we adjusted extensively for potential confounding variables, the possibility of residual confounding, such as maternal reproductive tract abnormalities and chromosomal abnormalities, cannot be completely ruled out. However, given that the study population comprised women who had a previous healthy live birth, the probability that these confounders had a substantial association with our results was low. Second, SA data were obtained by self-report; therefore, attrition bias and reporting bias might exist. In addition, some early cases of SA may be misidentified as delayed menstruation, leading to an underestimation of their incidence. Third, the prevalences of some SA risk factors (such as smoking, alcohol consumption, and thyroid disease) are relatively low in our population; hence, generalizing our results to other populations should be made with caution.

## Conclusions

Our results showed that an IPI of shorter than 18 months or an IPI of 36 months or longer after a healthy live birth was associated with an increased risk of subsequent SA. The recommended IPI range after a healthy live birth was 18 to 23 months. These findings are potentially valuable to clinicians when providing fertility counseling and to families when making a rational birth plan; these results may also facilitate the prevention of SA and improvement in neonatal outcomes.

## Notes

Supplement 1. **eAppendix.** IPI Grouping Method **eFigure 1.** Dose-Response Relationship Between Interpregnancy Interval and the Risk of Spontaneous Abortion in China From 2010 to 2020 **eTable 1.** Sensitivity Analyses in Using Different IPI Reference Groups When Assessing the Risk of Spontaneous Abortion in China From 2010 to 2020 **eTable 2**. Adjusted Odds Ratios of Spontaneous Abortion According to Interpregnancy Interval After Excluding Participants With History of Abortion in China From 2010 to 2020 **eTable 3**. Risks of Spontaneous Abortion According to Interpregnancy Interval in China From 2010 to 2020 (Reference Group: IPI 18-35 Months) **eTable 4**. Association Between the Interpregnancy Interval After Different Mode of Delivery and the Risk of Spontaneous Abortion in China From 2010 to 2020 (Reference Group: IPI 18-35 Months) **eTable 5**. Sensitivity Analysis Between the Interpregnancy Interval and the Risk of Spontaneous Abortion in China From 2010 to 2020 **eTable 6**. Sensitivity Analysis Between the Interpregnancy Interval and the Risk of Spontaneous Abortion in China From 2010 to 2020 (Reference Group: IPI 18-35 Months) **eFigure 2.** Odds Ratios for Spontaneous Abortion of Interpregnancy Interval Groups, According to Maternal Age at Previous Delivery **eFigure 3.** Miscarriage Rates in Different Interpregnancy Interval Groups Stratified by Prior Modes of Delivery

Supplement 2.
Data Sharing Statement
