# Supplementary material for: Interpregnancy Interval After Healthy Live Birth and Subsequent Spontaneous Abortion
Source: JAMA Netw Open. 2024 Jun 17;7(6):e2417397. doi: 10.1001/jamanetworkopen.2024.17397 (PMC11184457; doi:10.1001/jamanetworkopen.2024.17397)
Supplement: Supplement 1. — eAppendix. IPI Grouping Method eFigure 1. Dose-Response Relationship Between Interpregnancy Interval and the Risk of Spontaneous Abortion in China From 2010 to 2020 eTable 1. Sensitivity Analyses in Using Different IPI Reference Groups When Assessing the Risk of Spontaneous Abortion in China From 2010 to 2020 eTable 2. Adjusted Odds Ratios of Spontaneous Abortion According to Interpregnancy Interval After Excluding Participants With History of Abortion in China From 2010 to 2020 eTable 3. Risks of Spontaneous Abortion According to Interpregnancy Interval in China From 2010 to 2020 (Reference Group: IPI 18-35 Months) eTable 4. Association Between the Interpregnancy Interval After Different Mode of Delivery and the Risk of Spontaneous Abortion in China From 2010 to 2020 (Reference Group: IPI 18-35 Months) eTable 5. Sensitivity Analysis Between the Interpregnancy Interval and the Risk of Spontaneous Abortion in China From 2010 to 2020 eTable 6. Sensitivity Analysis Between the Interpregnancy Interval and the Risk of Spontaneous Abortion in China From 2010 to 2020 (Reference Group: IPI 18-35 Months) eFigure 2. Odds Ratios for Spontaneous Abortion of Interpregnancy Interval Groups, According to Maternal Age at Previous Delivery eFigure 3. Miscarriage Rates in Different Interpregnancy Interval Groups Stratified by Prior Modes of Delivery [file jamanetwopen-e2417397-s001.pdf]

Supplementary Online Content

Hu X, Yang Y, Wang L, et al. Interpregnancy interval after healthy live birth and subsequent spontaneous abortion. *JAMA Netw Open.* 2024;7(6):e2417397. doi:10.1001/jamanetworkopen.2024.17397

eAppendix. IPI Grouping Method
eFigure 1. Dose-Response Relationship Between Interpregnancy Interval and the Risk of Spontaneous Abortion in China From 2010 to 2020
eTable 1. Sensitivity Analyses in Using Different IPI Reference Groups When Assessing the Risk of Spontaneous Abortion in China From 2010 to 2020
eTable 2. Adjusted Odds Ratios of Spontaneous Abortion According to Interpregnancy Interval After Excluding Participants With History of Abortion in China From 2010 to 2020
eTable 3. Risks of Spontaneous Abortion According to Interpregnancy Interval in China From 2010 to 2020 (Reference Group: IPI 18-35 Months)
eTable 4. Association Between the Interpregnancy Interval After Different Mode of Delivery and the Risk of Spontaneous Abortion in China From 2010 to 2020 (Reference Group: IPI 18-35 Months)
eTable 5. Sensitivity Analysis Between the Interpregnancy Interval and the Risk of Spontaneous Abortion in China From 2010 to 2020
eTable 6. Sensitivity Analysis Between the Interpregnancy Interval and the Risk of Spontaneous Abortion in China From 2010 to 2020 (Reference Group: IPI 18-35 Months)
eFigure 2. Odds Ratios for Spontaneous Abortion of Interpregnancy Interval Groups, According to Maternal Age at Previous Delivery
eFigure 3. Miscarriage Rates in Different Interpregnancy Interval Groups Stratified by Prior Modes of Delivery

This supplemental material has been provided by the authors to give readers additional information about their work.

© 2024 Hu X et al. *JAMA Network Open.*

eAppendix. IPI Grouping Method

To rationalize the grouping of interpregnancy interval (IPI), we plotted the restricted cubic spline curve, it showed IPI and spontaneous abortion (SA) have a J-shaped curve relationship (χ^2 = 45.31; nonlinear P<0.001) (**eFigure 1**). The intersection of the curve with OR = 1 corresponds to IPI values of approximately 21.74 and 24.63 months.

© 2024 Hu X et al. *JAMA Network Open.*

eFigure 1. Dose-Response Relationship Between Interpregnancy Interval and the Risk of Spontaneous Abortion in China from 2010 to 2020

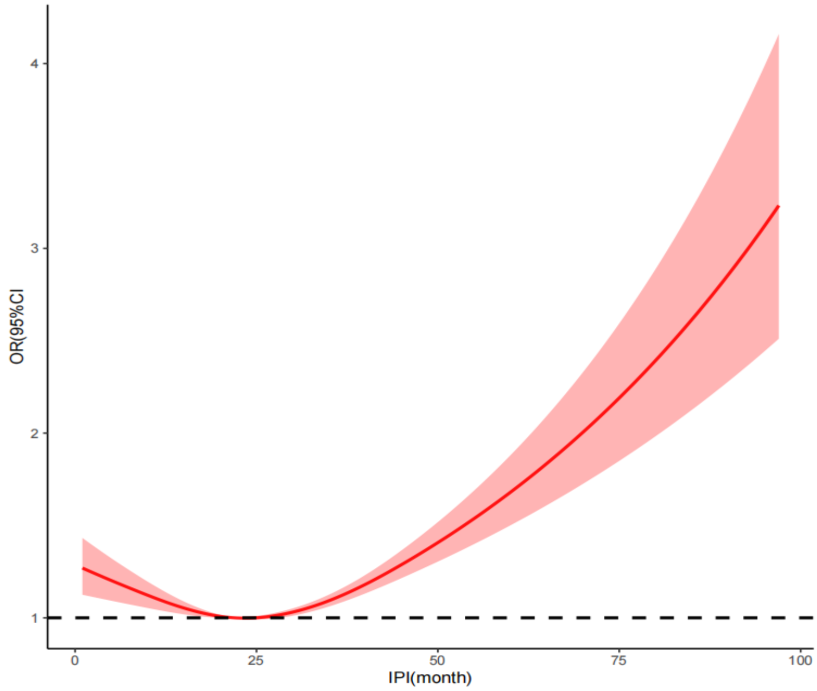

Abbreviation: OR = Odds Ratio; CI = confidence interval; IPI = Interpregnancy Interval.

Legend: OR was fully adjusted by maternal age at last menstrual period, pre-pregnancy body mass index, mode of previous delivery, alcohol consumption, smoking, history of abortion, maternal education level, and ethnicity. The line and shaded red areas represent ORs and 95% CIs, respectively. The black dashed lines demonstrate the reference level (OR=1)

An IPI of 18 to 23 months was used as the reference category in accordance to previous literature, the changes in the risk of SA at each six-month period were analyzed by logistic regression in those with IPI <24 months and IPI ≥ 18 months, respectively. The results showed that IPI <18 months was associated with an increased risk of SA in the group of IPI<24 months; In contrast, in the group of IPI ≥18 months, we found a significant increase in the risk of SA only when only when IPI were more than 36 months, so we classified IPI into 5 categories :<18, 18-23, 24-35, 36-59, ≥60 months (**eTable 1**).

© 2024 Hu X et al. *JAMA Network Open.*

eTable 1. Sensitivity Analyses in Using Different IPI Reference Groups When Assessing the Risk of Spontaneous Abortion in China From 2010 to 2020

IPI (mo)	Spontaneous Abortion No. (%)	OR (95% CI)	
		Crude OR (95% CI)	Adjusted OR (95% CI)[a]
**IPI < 24**			
<6	172 (2.52)	1.21 (1.02-1.43)	1.30 (1.08-1.56)
6-11	570 (2.27)	1.08 (0.97-1.22)	1.13 (1.00-1.28)
12-17	845 (2.36)	1.13 (1.02-1.25)	1.15 (1.03-1.29)
18-23	615 (2.10)	1.00 (Reference)	1.00 (Reference)
**IPI ≥ 18**			
18-23	615 (2.10)	1.00 (Reference)	1.00 (Reference)
24-29	559 (2.17)	1.03 (0.92-1.16)	1.02 (0.90-1.15)
30-35	475 (2.39)	1.14 (1.01-1.29)	1.10 (0.97-1.26)
36-41	391 (2.66)	1.27 (1.12-1.45)	1.22 (1.06-1.40)
42-47	274 (2.84)	1.36 (1.18-1.58)	1.30 (1.12-1.52)
48-53	177 (2.71)	1.30 (1.09-1.54)	1.27 (1.06-1.52)
54-59	119 (3.25)	1.56 (1.28-1.91)	1.48 (1.20-1.83)
≥60	183 (4.85)	2.38 (2.01-2.81)	2.12 (1.76-2.55)

Abbreviation: OR = Odds Ratio; CI = confidence interval; IPI = Interpregnancy Interval.

[a] Adjusted for maternal age at last menstrual period, pre-pregnancy body mass index, previous mode of delivery, alcohol consumption, smoking, history of abortion, maternal education level and ethnicity.

© 2024 Hu X et al. *JAMA Network Open.*

eTable 2. Adjusted Odds Ratios of Spontaneous Abortion According to Interpregnancy Interval After Excluding Participants With History of Abortion in China From 2010 to 2020

IPI (mo)	Spontaneous abortion	OR (95% CI)	
	No. (%)	Crude	Adjusted[a]
<18	1465 (0.85)	1.12 (1.02-1.24)	1.15 (1.04-1.27)
18-23	565 (0.33)	1.00 (Reference)	1.00 (Reference)
24-35	934 (0.54)	1.07 (0.96-1.19)	1.05 (0.94-1.17)
36-59	886 (0.51)	1.36 (1.23-1.52)	1.31 (1.17-1.47)
≥60	171 (0.10)	2.49 (2.09-2.96)	2.27 (1.88-2.73)

Abbreviation: OR = Odds Ratio; CI = confidence interval; IPI = Interpregnancy Interval.

[a]Adjusted for maternal age at last menstrual period, pre-pregnancy body mass index, mode of previous delivery, alcohol consumption, smoking, history of abortion, maternal education level and ethnicity.

© 2024 Hu X et al. *JAMA Network Open.*

eTable 3. Risks of Spontaneous Abortion According to Interpregnancy Interval in China From 2010 to 2020 (Reference Group: IPI 18-35 Months)

IPI (mo)	Spontaneous abortion	OR (95% CI)	
	No. (%)	Crude	Adjusted[a]
<18	1587 (2.35)	1.07 (1.00-1.14)	1.05 (0.97-1.13)
18-35	1649 (2.20)	1.00 (Reference)	1.00 (Reference)
36-59	961 (2.78)	1.27 (1.17-1.38)	1.30 (1.19-1.42)
≥60	183 (4.85)	2.27 (1.94-2.65)	2.27 (1.92-2.68)

Abbreviation: OR = Odds Ratio; CI = confidence interval; IPI = Interpregnancy Interval.

[a]Adjusted for maternal age at last menstrual period, pre-pregnancy body mass index, mode of previous delivery, alcohol consumption, smoking, history of abortion, maternal education level and ethnicity.

© 2024 Hu X et al. *JAMA Network Open.*

eTable 4. Association Between the Interpregnancy Interval After Different Mode of Delivery and the Risk of Spontaneous Abortion in China From 2010 to 2020 (Reference Group: IPI 18-35 Months)

IPI (mo)[a]	Crude OR (95% CI)		Adjusted OR (95% CI)[b]	
	Vaginal delivery	Caesarean delivery	Vaginal delivery	Caesarean delivery
**<18**	1.11 (1.02-1.20)	2.02 (1.78-2.29)	1.09 (1.00-1.19)	1.99 (1.74-2.27)
**18-35**	1.00 (Reference)	1.63 (1.47-1.80)	1.00 (Reference)	1.65 (1.48-1.84)
**36-59**	1.17 (1.05-1.30)	2.07 (1.85-2.32)	1.18 (1.06-1.33)	2.18 (1.94-2.45)
**≥60**	2.08 (1.66-2.60)	3.28 (2.65-4.07)	2.02 (1.59-2.58)	3.48 (2.78-4.35)

Abbreviation: OR = Odds Ratio; CI = confidence interval; IPI = Interpregnancy Interval.

[a] 209 Participants were not included in the etable4 due to a lack of data on mode of delivery, and IPI of 18-35 months after vaginal delivery was set as the reference group

[b]Adjusted for maternal age at last menstrual period, pre-pregnancy body mass index, mode of previous delivery, alcohol consumption, smoking, history of abortion, maternal education level and ethnicity.

© 2024 Hu X et al. *JAMA Network Open.*

eTable 5. Sensitivity Analysis Between the Interpregnancy Interval and the Risk of Abortion in China From 2010 to 2020

IPI (mo)	Spontaneous abortion	OR (95% CI)	
	No. (%)	Crude	Adjusted[a]
**<20 weeks**			
<18	323 (0.48)	1.01 (0.83-1.23)	1.11 (0.90-1.37)
18-23	139 (0.47)	1.00 (Reference)	1.00 (Reference)
24-35	264 (0.58)	1.22 (0.99-1.50)	1.14 (0.92-1.42)
36-59	272 (0.79)	1.67 (1.36-2.05)	1.49 (1.19-1.85)
≥60	81 (2.15)	4.65 (3.53-6.13)	3.72 (2.75-5.02)
**20-28 weeks**			
<18	1264 (1.87)	1.15 (1.04-1.28)	1.16 (1.04-1.30)
18-23	476 (1.63)	1.00 (Reference)	1.00 (Reference)
24-35	770 (1.69)	1.04 (0.93-1.17)	1.02 (0.91-1.16)
36-59	689 (1.99)	1.24 (1.10-1.39)	1.21 (1.06-1.37)
≥60	102 (2.70)	1.71 (1.38-2.12)	1.56 (1.24-1.97)

Abbreviation: OR = Odds Ratio; CI = confidence interval; IPI = Interpregnancy Interval.

[a]Adjusted for maternal age at last menstrual period, pre-pregnancy body mass index, mode of previous delivery, alcohol consumption, smoking, history of abortion, maternal education level and ethnicity.

© 2024 Hu X et al. *JAMA Network Open.*

eTable 6. Sensitivity Analysis Between the Interpregnancy Interval and the Risk of Spontaneous Abortion in China From 2010 to 2020 (Reference Group: IPI 18-35 Months)

IPI (mo)	Spontaneous abortion	OR (95% CI)	
	No. (%)	Crude	Adjusted[a]
**<20 weeks**			
<18	323 (0.48)	0.89 (0.77-1.03)	1.03 (0.88-1.20)
18-35	403 (0.54)	1.00 (Reference)	1.00 (Reference)
36-59	272 (0.79)	1.47(1.26-1.72)	1.37 (1.16-1.61)
≥60	81 (2.15)	4.10(3.22-5.22)	3.40 (2.62-4.42)
**20-28 weeks**			
<18	1264 (1.87)	1.12 (1.04-1.22)	1.48 (1.05-1.25)
18-35	1246 (1.66)	1.00 (Reference)	1.00 (Reference)
36-59	689 (1.99)	1.21 (1.10-1.33)	1.19 (1.08-1.32)
≥60	102 (2.70)	1.67 (1.36-2.05)	1.54 (1.23-1.92)

Abbreviation: OR = Odds Ratio; CI = confidence interval; IPI = Interpregnancy Interval.

[a] Adjusted for maternal age at last menstrual period, pre-pregnancy body mass index, mode of previous delivery, alcohol consumption, smoking, history of abortion, maternal education level and ethnicity.

© 2024 Hu X et al. *JAMA Network Open.*

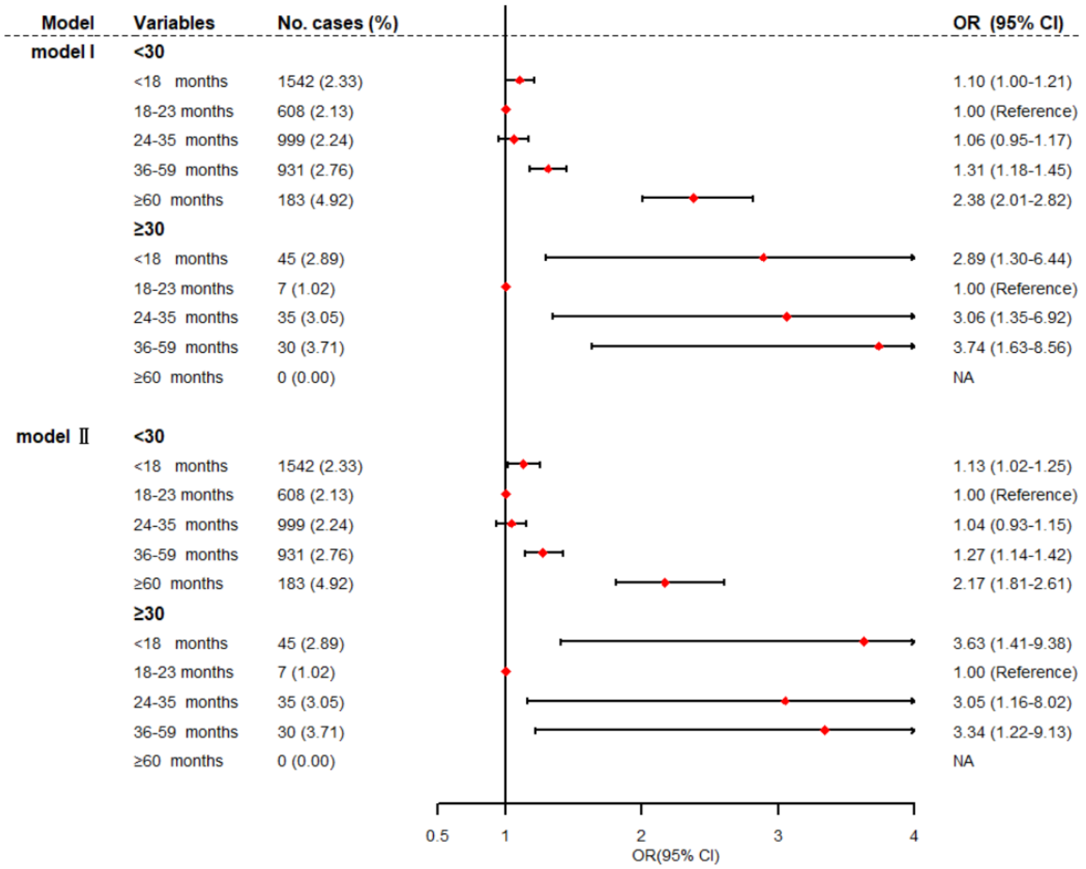

eFigure 2. Odds Ratios for Spontaneous Abortion of Interpregnancy Interval Groups, According to Maternal Age at Previous Delivery

Abbreviation: OR = Odds Ratio; CI = confidence interval; IPI = Interpregnancy Interval.

Model I: crude OR;

Model Ⅱ: OR was adjusted for pre-pregnancy body mass index, mode of previous delivery, alcohol consumption, smoking, history of abortion, maternal education level and ethnicity.

© 2024 Hu X et al. *JAMA Network Open.*

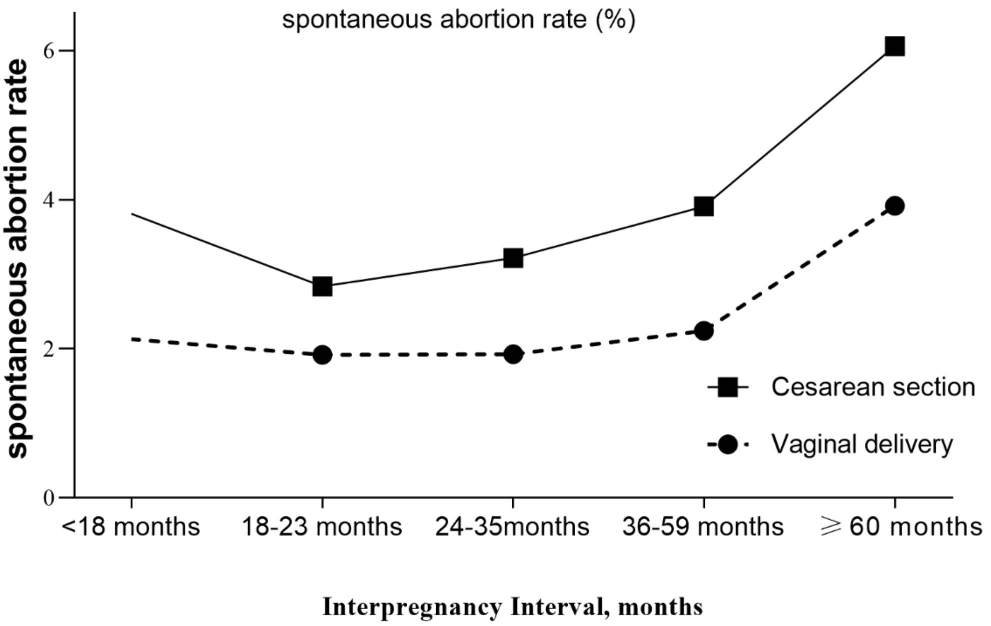

eFigure 3. Miscarriage Rates in Different Interpregnancy Interval Groups Stratified by Prior Modes of Delivery

© 2024 Hu X et al. *JAMA Network Open.*
