# Supplementary material for: Interpregnancy Interval After Healthy Live Birth and Subsequent Spontaneous Abortion
Source: JAMA Netw Open. 2024 Jun 17;7(6):e2417397. doi: 10.1001/jamanetworkopen.2024.17397 (PMC11184457; doi:10.1001/jamanetworkopen.2024.17397)
Supplement: Supplement 2. — Data Sharing Statement [file jamanetwopen-e2417397-s002.pdf]

Data Sharing Statement

Hu. Interpregnancy Interval After Healthy Live Birth and Subsequent Spontaneous Abortion. *JAMA Netw Open*. Published June 17, 2024. doi:10.1001/jamanetworkopen.2024.17397

Data
Data available: No
